# Predicting Drug Release From Degradable Hydrogels Using Fluorescence Correlation Spectroscopy and Mathematical Modeling

**DOI:** 10.3389/fbioe.2019.00410

**Published:** 2019-12-20

**Authors:** Saahil Sheth, Emily Barnard, Ben Hyatt, Muruhan Rathinam, Silviya Petrova Zustiak

**Affiliations:** ^1^Biomedical Engineering, Saint Louis University, St. Louis, MO, United States; ^2^Mathematics and Statistics, University of Maryland Baltimore County, Baltimore, MD, United States

**Keywords:** hydrogel, degradability, release, drug delivery, diffusion, computation

## Abstract

Predicting release from degradable hydrogels is challenging but highly valuable in a multitude of applications such as drug delivery and tissue engineering. In this study, we developed a simple mathematical and computational model that accounts for time-varying diffusivity and geometry to predict solute release profiles from degradable hydrogels. Our approach was to use time snapshots of diffusivity and hydrogel geometry data measured experimentally as inputs to a computational model which predicts release profile. We used two model proteins of varying molecular weights: bovine serum albumin (BSA; 66 kDa) and immunoglobulin G (IgG; 150 kDa). We used fluorescence correlation spectroscopy (FCS) to determine protein diffusivity as a function of hydrogel degradation. We tracked changes in gel geometry over the same time period. Curve fits to the diffusivity and geometry data were used as inputs to the computational model to predict the protein release profiles from the degradable hydrogels. We validated the model using conventional bulk release experiments. Because we approached the hydrogel as a black box, the model is particularly valuable for hydrogel systems whose degradation mechanisms are not known or cannot be accurately modeled.

## Introduction

Predicting release from degradable hydrogel networks is highly significant for a multitude of applications: designing multilayered or multicomponent drug delivery devices; controlling drug or other molecule delivery by pore size, pore size distribution, affinity, or other interactions (Lin and Metters, 2006), facilitating nutrient and gas exchange in three-dimensional cell scaffolds for tissue engineering (Leddy et al., 2004), controlling solute diffusivity via crowding and confinement to enhance extracellular matrix production by cells to create tissues *ex vivo* (Chen et al., 2011), and many others.

For example, hydrogels are at the forefront of an ever-growing drug delivery industry, estimated to become a $90 billion market by 2025 (Finn, 2016). Drug delivery methods range from oral, topical, intravenous, to—more recently, localized and targeted delivery. The increasing number and diversity of medications, such as small molecules, biologics, or cells, require more efficient and customizable delivery. Localized and controlled delivery approaches involving degradable hydrogels have proven particularly beneficial (Kurisawa et al., 2005; Bhattarai et al., 2010; Jain et al., 2017b).

Hydrogels are excellent candidates for drug delivery because of their biocompatibility, bioinertness, and ability to preserve the activity of biomacromolecules (Peppas et al., 1999; Lee and Mooney, 2001; Lutolf and Hubbell, 2003; Zustiak and Leach, 2011; Zustiak et al., 2013; Jain et al., 2017c). Degradable hydrogels are particularly advantageous because drug release can be controlled via degradation and there is no need to remove a device once the payload is depleted (Zhao and Milton Harris, 1998; Kim and Park, 2002). A common hydrogel used in drug delivery is polyethylene glycol (PEG), which is inherently non-degradable. However, various degradable moieties in the form of reactive end groups may be added to PEG to make it degradable (Lutolf and Hubbell, 2003; Zustiak et al., 2013; Jain et al., 2017a). Additionally, PEG can be synthesized with various chain lengths and multifunctionalities to further control degradation (Jain et al., 2017a).

While degradable hydrogels are increasingly sought, most studies developed to predict solute release from hydrogels focus on non-degradable polymeric matrices, or for simplicity ignore degradation and the associated changes in release. For example, some studies have focused on a “moving boundary problem,” where hydrogels are swellable (Brazel and Peppas, 2000; Fu and Soboyejo, 2011). However, these hydrogels, although swellable, do not degrade and thus the model does not fully predict release. There are also studies on predictive release from oral delivery systems such as hydroxypropyl methylcellulose compacted to form a tablet (Siepmann et al., 1999; Siepmann and Peppas, 2012). However, these systems are based on surface dissolution rather than bulk degradation, as would be the case of a hydrogel matrix. In another example, Mason et al. (2001) developed an exponential analytical model for release of model protein bovine serum albumin (BSA) from a degradable PEG-polylactic acid co-polymer hydrogel. However, this model is limited to the hydrogel described in the study, as one of the input model parameters depended on experimental measurements of hydrogel degradation products.

In this study, we developed a mathematical model to predict solute release profiles from degradable hydrogels using a simple, analytical approach based on a Fickian diffusion model. We experimentally measured diffusivity of two model proteins, namely BSA and immunoglobulin G (IgG) using fluorescence correlation spectroscopy (FCS) and the thickness of a PEG hydrogel over 5 h. These components were then input to a MATLAB algorithm to extract a predicted release profile. The predicted profile was compared to a release profile obtained using traditional bulk release methods and showed good agreement. We further discuss simplifications to the model, which could be implemented without sacrificing prediction accuracy.

## Theoretical and Computational Model

**Figure 1:**
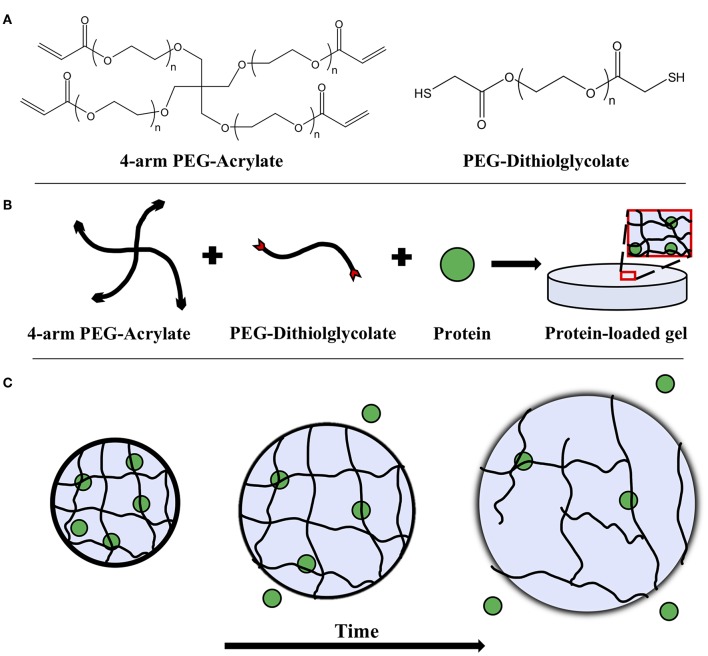
**(A)** Chemical structures of 4-arm PEG-acrylate macromer and the PEG-dithiolglycolate crosslinker. Schematic depicting **(B)** the formation of a PEG hydrogel with an embedded protein solute and **(C)** its degradation over time. The embedded protein is entrapped within the mesh network of the gel and as bulk degradation occurs, the mesh size of the hydrogel increases, allowing the proteins to diffuse more freely.

We assumed that the mechanism by which a protein solute is released from the hydrogel is Fickian diffusion. Due to the flat geometry of the hydrogel slab, a 1D partial differential equation (PDE) model for the concentration *c* of the protein was deemed adequate. This led to the PDE:

(1) $$\begin{array}{r} \frac{\partial c}{\partial t}=D\left(t\right)\frac{\partial^{2}c}{\partial x^{2}} \end{array}$$

where *D*(*t*) is the time-varying diffusivity of the protein solute. The diffusivity is time-varying since, as the hydrogel degrades with time (see Figure 1 for a schematic of bulk degradation), the mesh size increases, likewise increasing diffusivity.

In addition to diffusivity, the geometry of the gel slab is also time-varying as the gel degrades. This is reflected in the time-varying spatial domain of the PDE, which is −*h*(*t*) ≤ *x* ≤ *h*(*t*), where *h*(*t*) is the time-dependent half-thickness of the gel.

The PDE was subject to the boundary conditions, where solute concentration was assumed to be zero at gel boundaries, *c*(−*h*(*t*), *t*) = 0 = *c*(*h*(*t*), *t*). This assumption was based on the fact that the releasate solution surrounding the hydrogel was stirred during bulk release experiments (preventing formation of concentration gradients), and gel volume was ~100-times smaller than the volume of the releasate solution (creating an infinite sink environment). In addition, the PDE was subject to an initial condition, where the protein solute was uniformly distributed throughout the gel at *t* = 0, *c*(*x*, 0) = *c*_0_, −*h*(0) ≤ *x* ≤ *h*(0). To simplify the time-varying geometry of the gel, a change of variables of the form $y=\frac{x}{h\left(t\right)}$ was used to transform the domain from −*h*(*t*) ≤ *x* ≤ *h*(*t*) to −1 ≤ *y* ≤ 1, changing the PDE in Equation (1) to:

(2) $$\begin{array}{r} \frac{\partial c}{\partial t}=\frac{D\left(t\right)}{h^{2}\left(t\right)}\frac{\partial^{2}c}{\partial y^{2}}+\frac{h^{\prime }\left(t\right)}{h\left(t\right)}y\frac{\partial c}{\partial y} \end{array}$$

Subsequently, release rate $\dot{Q}\left(t\right)$ was formulated in terms of the concentration of the protein solute as:

(3) $$\begin{array}{r} \dot{Q}\left(t\right)=\;-\frac{\partial }{\partial t}\int_{-1}^{1}c\left(y,t\right)\;dy=\;-\int_{-1}^{1}\frac{\partial c\left(y,t\right)}{\partial t}\;dy \end{array}$$

The solution of the PDE model requires knowing *D*(*t*) and *h*(*t*) as functions of time. This can be accomplished in two ways. One approach is to develop a theoretical model to predict *D*(*t*)and *h*(*t*). This involves modeling how the hydrogel swells and degrades in time and how this in turn affects evolution of mesh size as well as hydrogel geometry as a function of time. Knowledge of mesh size at a given time may then be used to predict diffusivity at that time. See (Donovan et al., 2016) for instance on how to relate mesh size to diffusivity, described by us previously. However, reliably modeling the gel degradation is challenging. An alternative approach is to measure *D*(*t*) and *h*(*t*) at certain snapshots in time and then use curve fitting to obtain these as continuous functions of time for input to the PDE model. In this work, we tested this second approach.

To obtain continuous functions *D*(*t*) and *h*(*t*) from time snapshots of the solute diffusivity (measured by FCS) and the hydrogel slab thickness, time snapshot data can be fit with splines or some other family of curves using a weighted least-squares method. We decided to try using both quadratic splines as well as the exponential family:

(4) $$\begin{array}{r} f\left(t\right)=a-b\;e^{-ct} \end{array}$$

determined by three positive parameters, namely *a, b*, and *c*.

The numerical solution of the PDE in Equation (2) was accomplished as follows. First a spatial discretization using centered finite difference approximations was employed to obtain a system of ordinary differential equations (ODEs). Then this system was solved via the MATLAB ODE solver *ode23s*. The spatial integration in Equation (3) was approximated by Simpson's rule, and $\dot{Q}\left(t\right)$ was integrated in time with the MATLAB ODE solver *ode23s* along with the system of ODEs. Software was developed in MATLAB for the weighted least-squares fit with splines and the *lsqnonlin* program in MATLAB was used for the weighted least-squares fit with the exponential family.

To test whether the time-varying nature of diffusivity and hydrogel thickness affected release profiles, we also computed release profile predicted by the PDE model corresponding to constant *D-* and *h*-values, where the constant values were calculated as an average of all time points. Moreover, we decided to assess feasibility of predicting release profiles from measurements based on far fewer time snapshots. We only used the FCS measured diffusivities and hydrogel thickness measurements obtained at three carefully chosen time points, fitted the exponential family of curves for *D*(*t*) and *h*(*t*), and used these as inputs to the PDE model to compute predicted release.

## Materials and Methods

### Materials

4-arm PEG-acrylate (4-arm PEG-Ac; 10 kDa) was obtained from Laysan Bio Inc. (Arab, AL). The dithiol crosslinker PEG-dithiolglycolate (PEG-DD1; 3.4 kDa) was synthesized via Fischer esterification reaction as reported by us previously (Zustiak and Leach, 2010; Jain et al., 2017a). Fluorescent Dye Removal Columns were purchased from Thermo Scientific (Waltham, MA). Perfusion chambers were purchased from Sigma Aldrich (St. Louis, MO). Silicone isolator sheets (0.5 mm thick) from Grace Bio Labs (Bend, OR) were used as spacers. Bradford protein assay was obtained from Bio-Rad (Hercules, CA). BSA (66 kDa), Immunoglobulin G (IgG; 150 kDa), Atto 655-NHS ester, and all other reagents were procured from Millipore Sigma (St. Louis, MO) unless stated otherwise.

### Hydrogel Preparation

Hydrolytically degradable hydrogels were prepared by combining 4-arm PEG-Ac and the PEG-DD1 crosslinker in an equimolar ratio of reactive groups Ac:SH. A hydrogel matrix was then formed via Michael-type addition—a highly specific and mild gelation chemistry—between the reactive Ac and SH groups. Briefly, 4-arm PEG-Ac and PEG-DD1 were dissolved in a 0.3 M, pH 7.4 triethanolamine (TEA) buffer to make a 10% w/v PEG hydrogel precursor solution (Figures 1A,B). The precursor solution was then pipetted onto a parafilm-covered glass slide in 50 μL droplets. One millimeter thick silicon spacers were placed at the ends of the glass slide and a second parafilm-covered glass slide was used to sandwich the droplets to form thin slabs. The slabs were incubated at room temperature for 30 min to ensure complete gelation. Note that gelation time for gels made with 4-arm PEG-Ac and PEG-DD1 crosslinker at pH 7.4 is ~2 min (Jain et al., 2017a).

### Measurement of Hydrogel Swelling Ratio and Mesh Size

Hydrogels were characterized for swelling ratio (*Q*_*M*_) and mesh size (ξ) as a function of time. Hydrogel mass was measured immediately after fabrication to obtain the relaxed mass (*M*_*R*_). Afterwards, gels were stored in phosphate buffered saline (PBS) and re-weighed at 2, 4, and 6 h to obtain the swollen mass (*M*_*S*_). Gels were then placed in an oven at 60°C overnight and re-weighed to obtain the dry mass (*M*_*D*_). *Q*_*M*_ was calculated at the time points mentioned above as *M*_*S*_*/M*_*R*_. ξ was calculated according to the Flory-Rehner theory as follows (Canal and Peppas, 1989; Zustiak and Leach, 2011):

(5) $$\begin{array}{r} \xi =\;{\left(\upsilon_{2,s}\right)}^{-\frac{1}{3}}{\left(\frac{2C_{n}{\bar{M}}_{c}}{M_{r}}\right)}^{\frac{1}{2}}l \end{array}$$

where υ_2,*s*_ is the polymer volume fraction in the swollen state, *C*_*n*_ is the characteristic ratio for PEG, *M*_*C*_ is the average molecular weight between two adjacent crosslinks, *M*_*r*_ is the molecular weight of a PEG repeat unit, and *l* is the average bond length between the C-C and C-O bonds in a PEG repeat unit.

Note that the Flory-Rehner theory was developed for equilibrium swollen gels. Here, equilibrium swelling could not be established as the gels swelled continuously with degradation. Nevertheless, we expect the theory gives good indication of how ξ changed with time.

### Measurement of Hydrogel Geometry

Hydrogels were placed in microcentrifuge tubes with 1x PBS for swelling. At specified time points gels were removed from PBS and the diameters were measured using calipers, while gel thickness was measured using a micrometer. Change in hydrogel geometry upon swelling is depicted in Figure 1C.

### Measurement of Protein Bulk Release and Diffusivity

Hydrogels were prepared as mentioned previously with the following modification: 2% w/v of protein (BSA or IgG) was encapsulated by adding it directly to the hydrogel precursor solution prior to gelation (Figure 1B). For release experiments, the fabricated protein-loaded gels were transferred to a centrifuge tube with 5 mL of pre-warmed 1x PBS and placed on a shaker platform (Labquake Shaker, ThermoFisher, Waltham, MA) in an incubated environment at 37°C. A 1 mL releasate sample was taken at specific time points until degradation and replaced with 1 mL of fresh PBS to maintain a sink volume of 5 mL. The releasates were stored at −20°C until all time points were collected. Releasates' protein content was determined using Bradford protein assay following the manufacturer's protocol. An effective bulk diffusion coefficient (*D*_*e*_) was calculated using the following relation for short release times (*M*_*i*−_/*M*_∞_ < 0.6) (Ritger and Peppas, 1987):

(6) $$\begin{array}{r} \frac{M_{i}}{M_{\infty }}≅2{\left[\frac{D_{e}t}{\pi \delta^{2}}\right]}^{1/2} \end{array}$$

where *M*_*i*_ is protein content at each time point, *M*_∞_ is protein content at degradation, *t* is time, and δ is the half-thickness of the gel slab.

### Measurement of Protein Diffusivity Using Fluorescence Correlation Spectroscopy

For FCS measurements proteins were labeled with Atto 655-NHS ester fluorophore as per the manufacturer's protocol. Unbound fluorophores were removed using Fluorescent Dye Removal Columns as per the manufacturer's protocol with 95% efficiency. For FCS measurements, all gels were loaded with fluorescently labeled protein as described previously. Hydrogels were placed in a solution of Atto 655-labeled protein with the same concentration as in the gel (2% w/v) for the entirety of the experiment to avoid concentration gradients. At specific time points, hydrogels were removed from the soaking solution, gently blotted to remove excess solution, and placed on a #1 coverslip for FCS measurements. To avoid evaporation during measurement, a few drops of the soaking solution were pipetted on top of the gel, which was then covered with a custom lid.

The FCS instrument was calibrated using 0.2 nM Atto 655 dye in PBS. A 648 nm ps pulsed laser was used at an optical power of ~11.4 μW. A 640/LP filter was used along with a 655 dichroic mirror to obtain measurements for 120 s per sample. A minimum of 3 measurements at different locations were performed per hydrogel.

The obtained autocorrelation function *G(*τ*)* for each measurement was fitted by the following equation (Magde et al., 1974):

(7) $$\begin{array}{r} \text{G}\left(\tau \right)=1+\;\frac{1}{N}\frac{1}{\left[1+\left(\frac{\tau }{\tau_{D}}\right)\right]}\frac{1}{{\left[1+p\left(\frac{\tau }{\tau_{D}}\right)\right]}^{0.5}} \end{array}$$

where *N* is the number of fluorescent particles, τ_*D*_ is the diffusion time, *p* = *r*_*o*_*/z*_*o*_ is an instrumental constant, *r*_*o*_ is the radius of the focused laser beam spot, and *z*_*o*_ is the axial length of the focused laser beam spot.

For two non-interacting, diffusing solutes Equation (7) can be rewritten as (Michelman-Ribeiro et al., 2007; Zustiak et al., 2010):

(8) $$\begin{array}{r} G\left(\tau \right)=1+\;m_{1}\frac{1}{\left[1+\left(\frac{\tau }{\tau_{1}}\right)\right]}\frac{1}{{\left[1+p\left(\frac{\tau }{\tau_{1}}\right)\right]}^{0.5}}\;+\\ \;m_{2}\frac{1}{\left[1+\left(\frac{\tau }{\tau_{2}}\right)\right]}\frac{1}{{\left[1+p\left(\frac{\tau }{\tau_{2}}\right)\right]}^{0.5}} \end{array}$$

where *m*_1_ and *m*_2_ are related to the quantum yield and average number of each diffusing species and τ_1_ and τ_2_ are their respective diffusion times.

Here, the two-component autocorrelation function provided a more accurate fit as the gel precursor solutions were prepared with protein solutes that were exogenously labeled. Hence, they contained some un-reacted fluorophores in addition to fluorophore-labeled proteins. Note that τ_*D*_ for the free fluorophore was measured separately (and determined from the single component fit in Equation 7) and used as a fitted parameter (τ_2_) in Equation (8) to determine the diffusion time of the protein (τ_1_). Additionally, the autocorrelation function was fit using a Triplet Extended (3D) model to account for the possible excitation of molecular triplet states at higher laser intensities. Lastly, the autocorrelation function was normalized as follows:

(9) $$\begin{array}{r} Normalized\;G\left(\tau \right)=\frac{G\left(\tau_{D}\right)}{G\left(\tau_{0}\right)} \end{array}$$

where *G(*τ_*D*_*)* is the value of the Equation (8) at each time point and *G(*τ_0_*)* is the value of Equation (8) at the initial time point. The effective tracer diffusion coefficient for each protein was calculated from τ_D_ as (Zustiak et al., 2010):

(10) $$\begin{array}{r} D^{*}=\frac{{\left(r_{0}\right)}^{\text{2}}}{4\tau_{D}} \end{array}$$

### Statistical Analysis

Results are reported as mean averages with error bars of ± one standard deviation of triplicate samples from three independent experiments. A two-tailed Student's *t*-test was used to compare two samples. Differences between data sets were considered significant when *p* < 0.05.

## Results

### Hydrogel Swelling and Mesh Size as a Function of Time

As expected, the *Q*_*M*_ and mesh size of the hydrogel continuously increased with degradation (Table 1). Note that degradation was complete at ~8 h; however, at ~7 h the gels lose their shape and start fracturing. Hence, all measurements were conducted until 6 h, when the gels still had sufficient integrity to be handled. The *Q*_*M*_ increased by 7 times over the 6-h measurement. Note that for similar but non-degradable PEG gels, equilibrium swelling is achieved around 2 h (Zustiak et al., 2010). The lack of swelling saturation and the continuous increase in *Q*_*M*_ indicate continuous bulk degradation. Similarly, mesh size increased continuously by 1.3-times over the course of the measurement.

**Table 1:** Hydrogel characterization properties over the lifetime of the hydrogel. Errors represent one standard deviation.

**Time (h)**	**Swelling Ratio, *Q_**M**_***	**Mesh Size, ξ (nm)**
0	6.3 ± 0.2	—
2	27.7 ± 0.9	13.4 ± 0.3
4	36.1 ± 4.4	15.6 ± 1.0
6	44.7 ± 1.8	17.4 ± 0.2

### Measurements of Solute Diffusivity Using FCS

**Figure 2:**
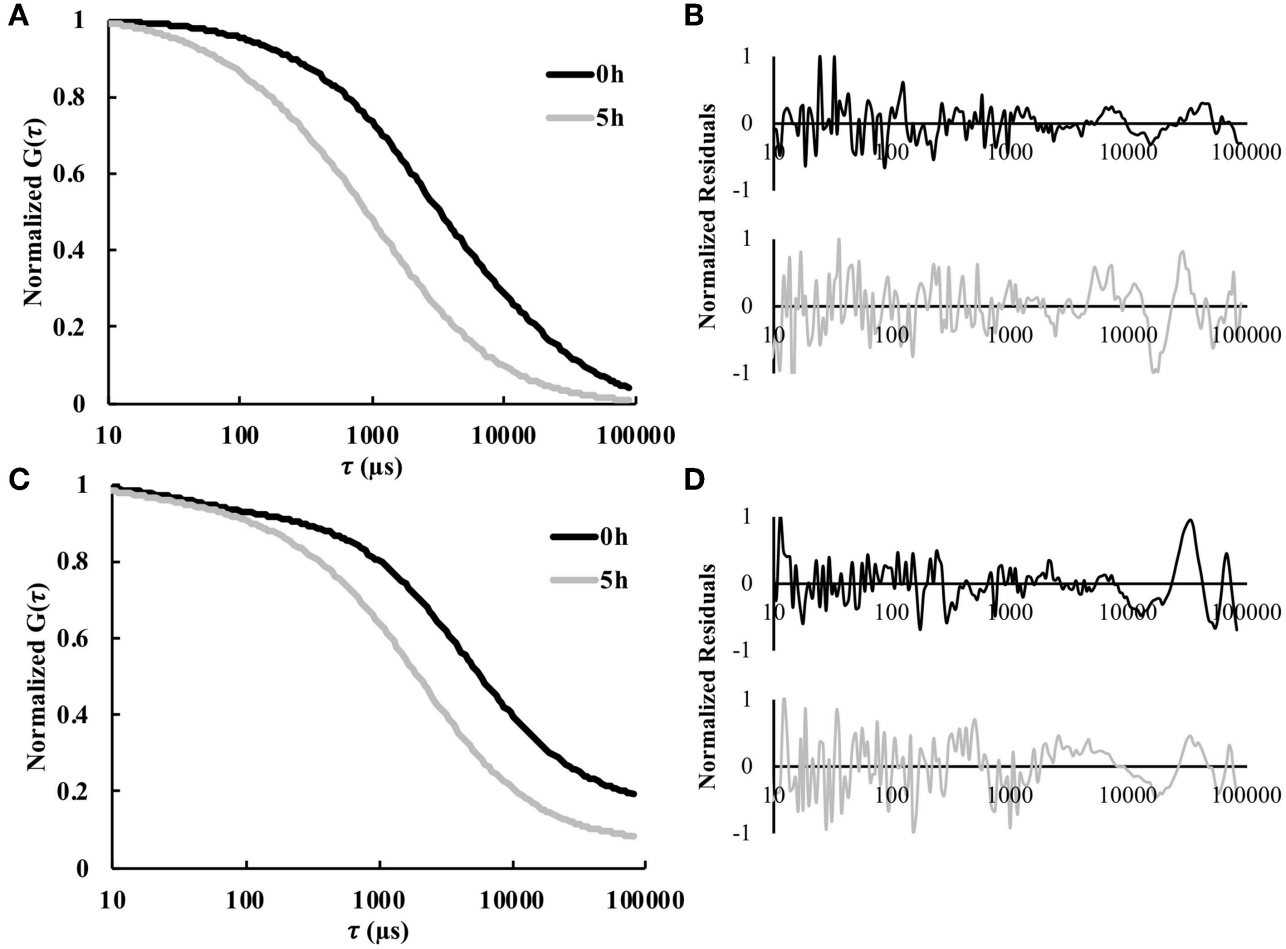
Representative normalized autocorrelation curves 0 and 5 h for fluorescently labeled **(A)** BSA and **(C)** IgG. The normalized residual curves indicate agreement between the autocorrelation curve and the fit for **(B)** BSA and **(D)** IgG (*n* = 9).

FCS was used to enable real-time *in situ* measurements of solute diffusivity in the hydrogel as a function of time. Figure 2 shows representative autocorrelation curves for both labeled model protein solutes used in this study, namely BSA and IgG. As expected, for both proteins the autocorrelation curves at 5 h were shifted to the left compared to the curves for 0 h, indicating substantially smaller diffusion times at the later time points (Figures 2A,C). The residuals indicate an excellent agreement between the raw autocorrelation data and the fit (Equation 8). Overall, diffusion times were higher at all time points for the larger IgG protein, compared to the smaller BSA. For BSA, the τ_*D*_-values were 3.3 ± 1.3 ms at 0 h and 1.4 ± 0.5 ms at 5 h. For IgG, the τ_*D*_-values were 4.2 ± 0.7 ms at 0 h and 2.2 ± 0.5 ms at 5 h. Note that τ_*D*_ is inversely proportional to diffusivity.

### Measurements of Bulk Release and Diffusivity

**Figure 3:**
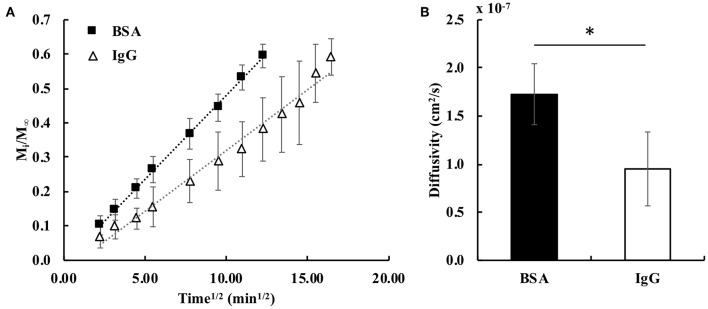
**(A)** Fractional release profiles of BSA and IgG from PEG hydrogels for small diffusion times (M_i_/*M*_∞_ < 0.6). The dotted lines represent the trendlines for the fractional release profiles of BSA (*R*^2^ = 0.999) and IgG (*R*^2^ = 0.983) (*n* = 6). **(B)** Calculated bulk effective diffusivities for BSA and IgG. A student's two-tailed *t*-test was performed. ^*^Designates significant differences (*p* < 0.05, *n* = 6).

The fractional bulk release profiles for both proteins embedded in the hydrogel are shown in Figure 3A. BSA released faster from the gel than IgG, exemplified by the higher slope of the BSA fractional release profile. This was expected, because BSA is smaller (hydrodynamic radius = 3.5 nm) than IgG (hydrodynamic radius = 4.7 nm) (Zustiak et al., 2010). Both proteins' hydrodynamic radii are smaller than the mesh size of the hydrogel (13–17 nm; Table 1), facilitating release. Calculation of the bulk diffusion coefficients using Equation (6) showed the *D*_*e*_ for BSA to be 1.8-times higher than that for IgG (Figure 3B).

### Validation of the Mathematical Model With Experimental Data

**Figure 4:**
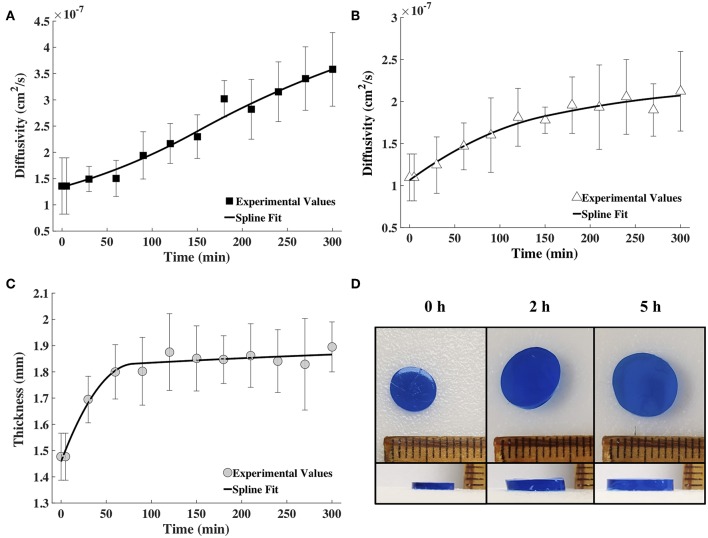
The spline fits calculated using MATLAB for the diffusion coefficient values obtained via FCS for fluorescently labeled **(A)** BSA and **(B)** IgG, and **(C)** the measured hydrogel thickness as a function of time (*n* = 9). **(D)** Images showing the increase in diameter and thickness of the hydrogel with time (blue coloring was used for visualization).

Tracer diffusivities (measured by FCS and computed from Equation 8) of labeled BSA and IgG continuously increased with gel degradation (Figures 4A,B). Diffusivity of labeled BSA exhibited a higher increase of 2.63-times during the 5 h of measurements, while the diffusivity of labeled IgG increased 1.93-times. Gel thickness increased 1.28 times (Figures 4C,D). The increase in thickness was abrupt in the first hour but mostly unchanged for the rest of the measurement time. Note that the gel also increased in diameter upon swelling. However, change in diameter was not used in the model, which was based on a 1D diffusion as explained earlier.

**Figure 5:**
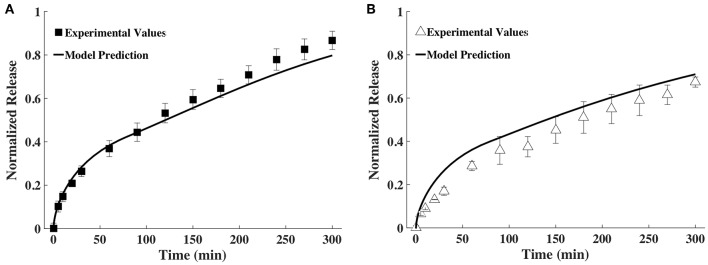
Comparison of release profiles of **(A)** BSA and **(B)** IgG obtained from the mathematical model and bulk release experiments (*n* = 9).

The time snapshots of the diffusivity values acquired via FCS measurements and the measured hydrogel thickness values were both fitted with splines as well as the exponential family in Equation (4). Quadratic splines with break points at 0, 80 and 300 min were used to fit thickness data, while quadratic splines with break points at 0, 150, and 300 min were used to fit diffusivity data. Note that the break points chosen were far fewer than the data points. This was done to obtain smooth curves. Since available MATLAB programs did not allow for choosing the break points to differ from the data points, a MATLAB program was written for this purpose (Barnard, 2018). Exponential fit was accomplished using the *lsqnonlin* program in MATLAB. Results were input to the mathematical model to obtain release profiles. Figure 5 shows only the results obtained via the spline fit, as both the spline fit and the exponential fit gave similar results (Figure S1). The model-computed release profile of BSA followed the experimental values very well until 90 min, beyond which point the model slightly under-predicted compared to the experimental release values. The model-computed release profile of IgG over-predicted at all time points compared to the experimental release values. However, the trend of the curve of the release profile was similar to the experimental values even with the over-prediction.

### Comparison of Time-Varying vs. Averaged Constant Diffusivity and Gel Height

**Figure 6:**
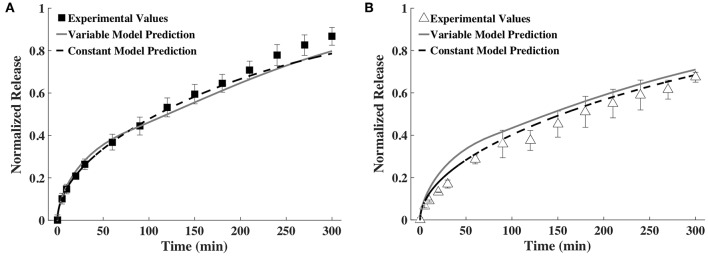
Release profiles calculated using a variable and a constant *D* and *h* model compared to experimental values of **(A)** BSA and **(B)** IgG (*n* = 9).

As explained earlier, the constant *D-* and *h*-values were calculated as an average of all time points. The constant average diffusivity for BSA was 2.43 × 10^−7^ and for IgG was 1.72 × 10^−7^ (both in cm^2^/s) and constant average gel thickness was 1.80 mm. The comparison of release profiles predicted using time-varying *D* and *h* as well as constant *D* and *h* against experimentally measured release profiles is shown in Figure 6. It can be seen that prediction from the constant model is quantitatively similar to that of the time-varying *D* and *h* model for BSA and somewhat different for IgG. In the case of IgG, the constant model better agreed with the experimental release profile.

### Prediction of Release Profile Based on Measurements at Three Time Points

**Figure 7:**
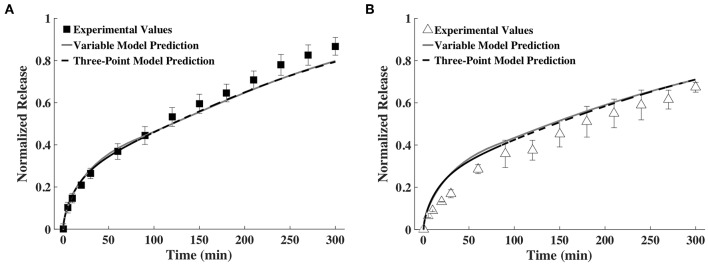
Release profiles calculated using a variable and a three-point *D* and *h* model compared to experimental values of **(A)** BSA and **(B)** IgG (*n* = 9).

To assess the feasibility of accurately predicting release profiles based on a small number of time snapshots, we decided to use the diffusivities and hydrogel thickness obtained at only three points in time. After some trial and error, we found that the release profiles predicted based on fitting the exponential family of curves for *D* and *h* measurements taken at 0, 60, and 240 min were very similar to the release profiles predicted by fitting splines for the data from all the time points. This is shown in Figure 7. We also tried fitting splines to the data from these time points. The results were not good and hence are not shown here.

## Discussion

Predicting release from degradable hydrogels has value in many applications such as drug delivery (Lin and Metters, 2006) and tissue engineering (Leddy et al., 2004). Degradable hydrogels are especially suitable for drug delivery because they don't require removal once the drug is delivered. In tissue engineering, they can degrade as native matrix is produced by cells. However, most studies developed to predict solute release from hydrogels focus on non-degradable polymeric matrices, or for simplicity ignore degradation and the associated changes in release (Brazel and Peppas, 2000; Mason et al., 2001; Fu and Soboyejo, 2011).

Here we chose to predict release from degradable hydrogels by focusing on the change in solute diffusivity and gel geometry as a function of time (both are caused by degradation). To monitor diffusivity changes, we could choose from two approaches—a mathematical model to predict the change in diffusivity with time or direct measurement. We measured directly, for better accuracy and because of the difficulties and errors associated with predicting variable solute diffusivity in hydrogels. While many models currently exist for predicting diffusivities in hydrogels, each has unique drawbacks and assumptions that could lead to inaccuracies (Davidson and Deen, 1988; Amsden, 1998a,b, 2002; Masaro and Zhu, 1999; Brazel and Peppas, 2000; Donovan et al., 2016). Also, models typically focus on predicting a constant diffusivity (as opposed to time-varying one as required in our approach) and require information on how degradation changes gel parameters such as mesh size. Our approach of using experimental data on diffusivity allowed us to treat the gel as a “black box.”

To measure diffusivity *in situ* as a function of time we used FCS. FCS measures the fluctuations emitted from a picoliter illuminated volume containing nanomolar concentrations of fluorescent solutes, typically induced by the motion of the solutes moving in and out of the volume. This powerful technique has been increasingly employed to assess solute diffusion in complex environments such as crowded media (Dauty and Verkman, 2004; Weiss et al., 2004; Zustiak et al., 2011), polymer gels (Michelman-Ribeiro et al., 2004; Stylianopoulos et al., 2010; Zustiak et al., 2010), and biological tissues (Lee et al., 2011). Importantly, being a single molecule technique, FCS can measure diffusivity of solutes at low concentrations with unique sensitivity and precision (Zustiak et al., 2010). The nanomolar working concentrations enable studies of rare and expensive solutes (such as growth factors), which could be cost-prohibitive for other approaches. For comparison, the bulk release experiments used here to validate the model, require mM solute concentrations and are labor-intensive and time-consuming.

However, while we chose FCS for measuring diffusivities, our approach is broader and only requires that a method is used to measure diffusivity as a function of time. New methods to measure diffusivities non-invasively in real time are continuously being developed, but one needs to be aware that each method has advantages and drawbacks. For example, dynamic light scattering (DLS) is well-established for measuring diffusivities of dilute solutes in small volumes of liquid or sol-gel samples (Joosten et al., 1990). However, it requires a solute and polymer of disparate sizes, since both contribute to the scattering signal. Hence, DLS is most suitable for measuring proteins, viruses, micellesk and other microparticles, but not for nanosolutes such as salts and small molecule drugs. DLS has also been shown to overestimate diffusion coefficients (Annunziata et al., 2005). Rayleigh interferometry has been suggested as a technique of superior accuracy, but requires macroscopic concentration gradients, which are not practical for many applications (Annunziata et al., 2005). Nuclear magnetic resonance microscopy (NMR) can measure diffusion of solutes in liquids or gels, but has limits related to spin-spin relaxation times, magnetic gradient strength and eddy currents, which could skew results (Massaro and Zhu, 1999). Further, NMR that allows studying solid samples also requires expensive and dedicated facilities not readily available to most investigators. Fluorescence recovery after photobleaching (FRAP) (Berk et al., 1993) has a great potential, but it is not suitable for low solute concentrations and has the additional drawback of occasional reversible photobleaching, which can skew diffusion results.

For this study we chose to use a PEG hydrogel that would degrade in a matter of hours, to more easily follow changes in solute diffusivity and gel thickness with gel degradation. Hence, we used a hydrogel formed from 4-arm PEG-Ac crosslinked with the custom-synthesized crosslinker PEG-DD1 (Figure 1), which degrades by hydrolysis in ~8 h (Jain et al., 2017a). Experiments were only conducted for 5 h, as the gel lost integrity and was hard to handle after that time. *Q*_*M*_ and mesh size continuously increased, as expected for a bulk degrading hydrogel (Table 1).

To underscore the utility of our approach, we tested two model proteins of varying sizes. Because BSA (66 kDa; *R*_*h*_ = 3.5 nm) is smaller than IgG (150 kDa; *R*_*h*_ = 4.7 nm), we expected BSA to diffuse and release from the gel more rapidly (Zustiak and Leach, 2011; Jain et al., 2017b). Because both proteins are smaller in hydrodynamic radius than the gel mesh (13–17 nm), a diffusion and degradation-controlled release was expected. The changes in these proteins' diffusivities as a function of hydrogel degradation was fitted via a spline or an exponential fit to obtain input to the mathematical model. The data showed a general trend of increasing diffusivity throughout degradation (Figures 4A,B), which was expected based on the increasing mesh size of the gel (Table 1). Further, in Figure 2, we noted that the 5 h timepoint curve was noticeably shifted to the left compared to the 0 h timepoint, indicating a smaller diffusion time (Zustiak et al., 2010; Jain et al., 2017b). There was a more pronounced shift for the smaller BSA compared to IgG; because IgG is larger it will not be able to diffuse as freely as BSA inside the hydrogel.

With the diffusivity and thickness data points, a curve of best fit was generated using MATLAB to be used as the input variables for the mathematical model (Figure 4). The gel thickness did increase ~29%, however the diameter also increased ~33%. We chose not to take the diameter of the gel into account because we are assuming 1D diffusion of the solute in the z-direction. Since the gel can be modeled as a thin cylinder, the gel's two faces are the only diffusing surfaces; as gel thickness increases, the path for diffusion also increases.

To validate the mathematical model, we compared its predictions to release profiles acquired through bulk release experiments. Although the mathematical model's predicted release profile was very similar to the experimental one, there were differences, especially at the later time points. For BSA, model predictions seemed to align well until after ~90 min, at which point the model began to slightly under-predict release (Figure 5A). However, model prediction appeared to be within the error range of the experimental values. With IgG, the model continuously over-predicted release, but the release trends aligned well (Figure 5B). We suggest over-prediction is due to the way diffusivity was measured via bulk release or through FCS. Overall, we noted that diffusivities measured by FCS (tracer diffusivity, Equation 10) were slightly higher than the ones obtained from bulk release experiments (bulk diffusivity, Equation 6). For example, the bulk diffusivity for BSA was 1.71 × 10^−7^ cm^2^/s, corresponding to the initial tracer diffusivity at <100 min (Figures 3B, 4A). However, for the larger IgG, the bulk diffusivity was 0.89 × 10^−7^ cm^2^/s compared to the tracer diffusivity, which started at >1 × 10^−7^ cm^2^/s (Figures 3B, **4B**). We expected to see similar, but not exactly the same, diffusivities obtained through FCS and bulk release experiments for several reasons. First, we expected similarities because of the way we performed the FCS measurements. With FCS we measured at multiple gel locations to ascertain that we were not measuring only local diffusivity, but that our measurements represented the entire gel. Further, the illuminated FCS volume had a diameter in the micrometer range, while the hydrogel was nanoporous (mesh size of ~13–17 nm). Hence, even the local diffusivities measured should represent the bulk hydrogel. However, it has also been shown that tracer (as measured by FCS) and bulk diffusivities could differ, with the tracer diffusivity being slightly higher than the bulk (Anderson and Reed, 1976). This could partially explain why, especially for IgG (Figure 4B), the model which relied on FCS data slightly over-predicted release compared to data from bulk release experiments. However, it is important to note that the model overall followed closely the trend predicted by bulk experiments.

We next explored ways to simplify the model or the experimental measurements required for input to the model, to make our approach more broadly accessible to others. Prediction accuracy was compared to the time-varying approach shown in Figure 5 for all cases. In one simplification, the diffusivity and thickness values were averaged across all time points and one constant value was used for each input (Figure 6). Using an averaged constant diffusivity and gel thickness (averaged over the entire process of gel degradation) simplifies the mathematical model used (the PDEs) and does not require a change in variable. For both BSA and IgG, the variable and constant model predictions were similar, with the constant model fitting the experimental data better. We believe the similarity between release predictions by the variable and the constant models may be partially explained by how *D* and *h* change as gel degrades. It must be noted that an increase in *D* increases the rate of release, while an increase in *h* decreases the rate of release. Since both *D* and *h* are increasing with time, the effects of these increases are somewhat but not entirely canceling each other. The constant model may sometimes better predict the release profile because the constant *D* and *h* are computed as averages over all time points. Thus, there is greater cancelation of experimental measurement errors, which improves accuracy of the values of *D* and *h* used.

In another simplification, we considered decreasing the number of points required to profile the change in diffusivity and gel thickness to only three. Reducing the number of time points simplifies experimental data acquisition. We found that fitting the exponential family of curves to the diffusivity and thickness data at 0, 60, and 240 min was sufficient to predict release profile, as shown in Figure 7. While a trial and error approach was used to select the points, we made sure to include the 0 min time point to represent the gel prior to swelling, a second early time point and one late time point.

Lastly, in fitting continuous smooth curves to the time snapshots to obtain *D*(*t*) and *h*(*t*), two approaches were explored: quadratic splines and an exponential family of curves given by Equation (4). The advantage of the splines is that the resulting weighted least squares minimization problem is linear, while the corresponding minimization problem for the exponential family is non-linear. The disadvantage of using splines is that they may yield an unrealistic oscillatory curve; this can be avoided with the exponential family. This is particularly useful in fitting the gel thickness *h*(*t*), as the derivative *h*′(*t*) is not expected to become negative. Moreover, using splines involves several choices which include the degree of the splines, degree of smoothness as well as choice of the break points. Figure S1 demonstrates that results of the spline fit and exponential fit are very close. When the three time points fit is used, the better method of fit is the exponential.

## Conclusions

We have developed a mathematical and computational model which uses time snapshots of diffusivity and geometry data as inputs to predict the release profile of proteins embedded in degrading hydrogels. The mathematical model based on Fickian diffusion was described by a 1D PDE with time-varying diffusivity and hydrogel thickness. The time snapshots of diffusivity were measured experimentally by FCS. The overall model was validated for the two model proteins BSA and IgG embedded in PEG hydrogels by comparing our model-predicted release profiles to conventional bulk release profiles obtained through experimentation. Our approach of predicting the release profile bypasses the difficult task of modeling gel degradation to predict time-varying diffusivity and geometry. Further, we showed that the model could be simplified further without loss of accuracy by using either an averaged constant diffusivity and gel thickness (for simpler mathematical modeling) or a three-point measurement (for simpler experimental measurements). The developed approach could be valuable in various applications of degradable hydrogels, such as drug delivery to predict release profiles; or tissue engineering to predict nutrient and metabolite exchange within scaffolds.

## Data Availability Statement

The datasets generated for this study are available on request to the corresponding author.

## Author Contributions

SZ and MR conceived, designed, and supervised the study. SS performed all experimental procedures. EB, BH, and MR developed and implemented the mathematical model. SZ, MR, and SS wrote the manuscript with input from all authors.

### Conflict of Interest

The authors declare that the research was conducted in the absence of any commercial or financial relationships that could be construed as a potential conflict of interest.

## References

[B1] Amsden B. (1998a). Solute diffusion in hydrogels.: an examination of the retardation effect. Poly. Gels Networks 6, 13–43. 10.1016/S0966-7822(97)00012-9

[B2] Amsden B. (1998b). Solute diffusion within hydrogels. Mech. Models Macromol. 31, 8382–8395. 10.1021/ma980765f

[B3] Amsden B. (2002). Modeling solute diffusion in aqueous polymer solutions. Polymer 43, 1623–1630. 10.1016/S0032-3861(01)00749-2

[B4] Anderson J. L. Reed C. C. (1976). Diffusion of spherical macromolecules at finite concentration. J. Chem. Phy. 64, 3240–3250. 10.1063/1.432664

[B5] Annunziata O. Buzatu D. Albright J. G. (2005). Protein diffusion coefficients determined by macroscopic-gradient rayleigh interferometry and dynamic light scattering. Langmuir 21, 12085–12089. 10.1021/la052147f 16342976

[B6] Barnard E. (2018). Modeling the Delivery of Drugs from a Water-Soluble Gel. Baltimore, MD: University of Maryland.

[B7] Berk D. A. Yuan F. Leunig M. Jain R. K. (1993). Fluorescence photobleaching with spatial fourier analysis: measurement of diffusion in light-scattering media. Biophys. J. 65, 2428–2436. 10.1016/S0006-3495(93)81326-2 8312481 PMC1225983

[B8] Bhattarai N. Gunn J. Zhang M. (2010). Chitosan-based hydrogels for controlled, localized drug delivery. Adv. Drug Deliv. Rev. 62, 83–99. 10.1016/j.addr.2009.07.019 19799949

[B9] Brazel C. S. Peppas N. A. (2000). Modeling of drug release from swellable polymers. Eur. J. Pharmaceut. Biopharmaceut. 49, 47–58. 10.1016/S0939-6411(99)00058-2 10613927

[B10] Canal T. Peppas N. A. (1989). Correlation between mesh size and equilibrium degree of swelling of polymeric networks. J. Biomed. Mater. Res. 23, 1183–1193. 10.1002/jbm.820231007 2808463

[B11] Chen C. Loe F. Blocki A. Peng Y. Raghunath M. (2011). Applying macromolecular crowding to enhance extracellular matrix deposition and its remodeling *in vitro* for tissue engineering and cell-based therapies. Adv. Drug Deliv. Rev. 63, 277–290. 10.1016/j.addr.2011.03.003 21392551

[B12] Dauty E. Verkman A. S. (2004). Molecular crowding reduces to a similar extent the diffusion of small solutes and macromolecules: measurement by fluorescence correlation spectroscopy. J. Mol. Recogn. 17, 441–447. 10.1002/jmr.709 15362103

[B13] Davidson M. G. Deen W. M. (1988). Hindered diffusion of water-soluble macromolecules in membranes. Macromolecules 21, 3474–3481. 10.1021/ma00190a022

[B14] Donovan P. Chehreghanianzabi Y. Rathinam M. Zustiak S. P. (2016). Homogenization theory for the prediction of obstructed solute diffusivity in macromolecular solutions. PLoS ONE 11:e0146093. 10.1371/journal.pone.0146093 26731550 PMC4701423

[B15] Finn M. (2016). Top Markets Report: Pharmaceuticals. US Department of Commerce, International Trade Administration, Industry.

[B16] Fu G. Soboyejo W. (2011). Investigation of swellable poly (N-isopropylacrylamide) based hydrogels for drug delivery. Mater. Sci. Eng. 31, 1084–1090. 10.1016/j.msec.2011.03.009

[B17] Jain E. Hill L. Canning E. Sell S. A. Zustiak S. (2017a). Control of gelation, degradation and physical properties of polyethylene glycol hydrogels through the chemical and physical identity of the crosslinker. J. Mater. Chem. 5, 2679–2691. 10.1039/C6TB03050E 32264047

[B18] Jain E. Sheth S. Dunn A. Zustiak S. P. Sell S. (2017b). Sustained release of multicomponent platelet-rich plasma proteins from hydrolytically degradable PEG hydrogels. J. Biomed. Mater. Res. Part A 105, 3304–3314. 10.1002/jbm.a.36187 28865187

[B19] Jain E. Sheth S. Polito K. Sell S. A. Zustiak S. (2017c). Storage stability of biodegradable polyethylene glycol microspheres. Mater. Res. Exp. 4:105403 10.1088/2053-1591/aa8e37

[B20] Joosten J. G. Geladé E. T. Pusey P. N. (1990). Dynamic light scattering by nonergodic media: brownian particles trapped in polyacrylamide gels. Phys. Rev. A 42:2161. 10.1103/PhysRevA.42.2161 9904264

[B21] Kim M. R. Park T. G. (2002). Temperature-responsive and degradable hyaluronic acid/pluronic composite hydrogels for controlled release of human growth hormone. J. Control. Rel. 80, 69–77. 10.1016/S0168-3659(01)00557-0 11943388

[B22] Kurisawa M. Chung J. E. Yang Y. Y. Gao S. Uyama H (2005). Injectable biodegradable hydrogels composed of hyaluronic acid–tyramine conjugates for drug delivery and tissue engineering. Chem. Commun. 34, 4312–4314. 10.1039/b506989k 16113732

[B23] Leddy H. A. Awad H. A. Guilak F. (2004). Molecular diffusion in tissue-engineered cartilage constructs: effects of scaffold material, time, and culture conditions. J. Biomed. Mater. Res. Part B Appl. Biomater. 70, 397–406. 10.1002/jbm.b.30053 15264325

[B24] Lee J. Masato S. Kiminori U. Mochida J. (2011). Measurement of diffusion in articular cartilage using fluorescence correlation spectroscopy. BMC Biotechnol. 11:19. 10.1186/1472-6750-11-19 21366913 PMC3061899

[B25] Lee K. Y. Mooney D. J. (2001). Hydrogels for tissue engineering. Chem. Rev. 101, 1869–1880. 10.1021/cr000108x 11710233

[B26] Lin C.-C. Metters A. T. (2006). Hydrogels in controlled release formulations: network design and mathematical modeling. Adv. Drug Deliv. Rev. 58, 1379–1408. 10.1016/j.addr.2006.09.004 17081649

[B27] Lutolf M. Hubbell J. (2003). Synthesis and physicochemical characterization of end-linked poly (ethylene glycol)-co-peptide hydrogels formed by michael-type addition. Biomacromolecules 4, 713–722. 10.1021/bm025744e 12741789

[B28] Magde D. Elson E. L. Webb W. W. (1974). Fluorescence correlation spectroscopy. II. an experimental realization. Biopolymers 13, 29–61. 10.1002/bip.1974.360130103 4818131

[B29] Masaro L. Zhu X. (1999). Physical models of diffusion for polymer solutions, gels and solids. Prog. Poly. Sci. 24, 731–775. 10.1016/S0079-6700(99)00016-7

[B30] Mason M. N. Metters A. T. Bowman C. N. Anseth K. (2001). Predicting controlled-release behavior of degradable PLA-b-PEG-b-PLA hydrogels. Macromolecules 34, 4630–4635. 10.1021/ma010025y

[B31] Massaro L. Zhu X. X. (1999). Self-diffuion of end-capped oligo(ethylene glycols) in poly(vinyl alcohol) aqueous solutions and gels. Macromolecules 32, 5383–5390. 10.1021/ma9902908

[B32] Michelman-Ribeiro A. Boukari H. Nossal R. Horkay F. (2004). Structural changes in polymer gels probed by fluorescence correlation spectroscopy. Macromolecules 37, 10212–10214. 10.1021/ma048043d

[B33] Michelman-Ribeiro A. Horkay F. Nossal R. Boukari H. (2007). Probe diffusion in aqueous poly (vinyl alcohol) solutions studied by fluorescence correlation spectroscopy. Biomacromolecules 8, 1595–1600. 10.1021/bm061195r 17441767

[B34] Peppas N. A. Keys K. B. Torres-Lugo M. Lowman A. M. (1999). Poly (ethylene glycol)-containing hydrogels in drug delivery. J. Control. Rele. 62, 81–87. 10.1016/S0168-3659(99)00027-9 10518639

[B35] Ritger P. L. Peppas N. A. (1987). A simple equation for description of solute release II fickian and anomalous release from swellable devices. J. Control. Rele. 5, 37–42. 10.1016/0168-3659(87)90035-6 25356469

[B36] Siepmann J. Kranz H. Bodmeier R. Peppas N. (1999). HPMC-matrices for controlled drug delivery: a new model combining diffusion, swelling, and dissolution mechanisms and predicting the release kinetics. Pharmaceut. Res. 16, 1748–1756. 10.1023/A:1018914301328 10571282

[B37] Siepmann J. Peppas N. (2012). Modeling of drug release from delivery systems based on hydroxypropyl methylcellulose (HPMC). Adv. Drug Deliv. Rev. 64, 163–174. 10.1016/j.addr.2012.09.028 11369079

[B38] Stylianopoulos T. Poh M. Z. Insin N. Bawendi M. G. Fukumura D. Munn L. L.. (2010). Diffusion of particles in the extracellular matrix: the effect of repulsive electrostatic interactions. Biophys. J. 99, 1342–1349. 10.1016/j.bpj.2010.06.016 20816045 PMC2931749

[B39] Weiss M. Elsner M. Kartberg F. Nilsson T. (2004). Anomalous subdiffusion is a measure for cytoplasmic crowding in living cells. Biophys. J. 87, 3518–3524. 10.1529/biophysj.104.044263 15339818 PMC1304817

[B40] Zhao X. Milton Harris J. (1998). Novel degradable poly (ethylene glycol) hydrogels for controlled release of protein. J. Pharmaceut. Sci. 87, 1450–1458. 10.1021/js980065o 9811505

[B41] Zustiak S. P. Boukari H. Leach J. B. (2010). Solute diffusion and interactions in cross-linked poly (ethylene glycol) hydrogels studied by fluorescence correlation spectroscopy. Soft Matter 6, 3609–3618. 10.1039/c0sm00111b 24282439 PMC3838862

[B42] Zustiak S. P. Leach J. B. (2010). Hydrolytically degradable poly (ethylene glycol) hydrogel scaffolds with tunable degradation and mechanical properties. Biomacromolecules 11, 1348–1357. 10.1021/bm100137q 20355705 PMC3050024

[B43] Zustiak S. P. Leach J. B. (2011). Characterization of protein release from hydrolytically degradable poly (ethylene glycol) hydrogels. Biotechnol. Bioeng. 108, 197–206. 10.1002/bit.22911 20803477 PMC3057087

[B44] Zustiak S. P. Nossal R. Sackett D. L. (2011). Hindered diffusion in polymeric solutions studied by fluorescence correlation spectroscopy. Biophys. J. 101, 255–264. 10.1016/j.bpj.2011.05.035 21723836 PMC3127197

[B45] Zustiak S. P. Pubill S. Ribeiro A. Leach J. B. (2013). Hydrolytically degradable poly (ethylene glycol) hydrogel scaffolds as a cell delivery vehicle: characterization of PC12 cell response. Biotechnol. Prog. 29, 1255–1264. 10.1002/btpr.1761 24474590 PMC3795798

