# Supplementary material for: Predicting Drug Release From Degradable Hydrogels Using Fluorescence Correlation Spectroscopy and Mathematical Modeling
Source: Front Bioeng Biotechnol. 2019 Dec 20;7:410. doi: 10.3389/fbioe.2019.00410 (PMC6951421; doi:10.3389/fbioe.2019.00410)
Supplement: Supplementary file 1 [file Table_1.DOCX]

**
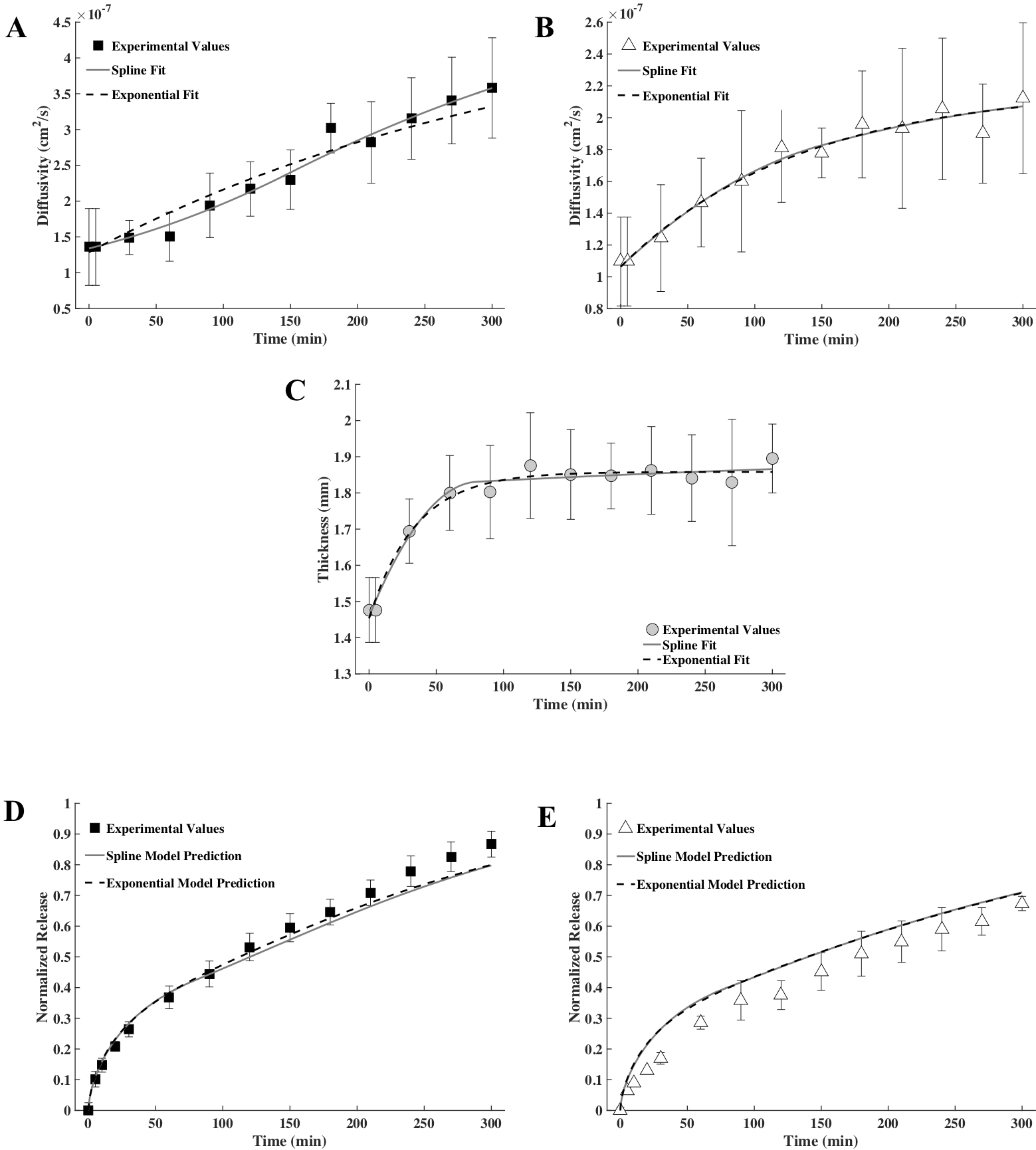
**

**Figure S1:** **Comparison of spline vs exponential data fit.** The fits calculated using MATLAB for the diffusion coefficient values obtained via FCS for fluorescently labeled **(A)** BSA and **(B)** IgG, and **(C)** the measured hydrogel thickness as a function of time. Comparison of release profiles of **(D)** BSA and **(E)** IgG obtained from the mathematical model and bulk release experiments.
